# Self-sorted photoconductive xerogels†
†Electronic supplementary information (ESI) available: Full experimental details, rheological data, p*K*_a_ titrations, further NMR data, full details of the photoconductivity set-up, further photoconductivity measurements. See DOI: 10.1039/c6sc02644c



**DOI:** 10.1039/c6sc02644c

**Published:** 2016-07-01

**Authors:** Emily R. Draper, Jonathan R. Lee, Matthew Wallace, Frank Jäckel, Alexander J. Cowan, Dave J. Adams

**Affiliations:** a Department of Chemistry , University of Liverpool , Crown Street , Liverpool , L69 7ZD , UK . Email: d.j.adams@liverpool.ac.uk; b Department of Physics , University of Liverpool , Oxford Street , Liverpool , L69 7ZE , UK; c Stephenson Institute for Renewable Energy , University of Liverpool , Peach Street , Liverpool , L69 7ZF , UK

## Abstract

We show that a perylene bisimide (PBI)-based gelator forms self-sorted mixtures with a stilbene-based gelator. To form the self-sorted gels, we use a slow pH change induced by the hydrolysis of glucono-δ-lactone (GdL) to gluconic acid. We prove that self-sorting occurs using NMR spectroscopy, UV-Vis spectroscopy, rheology, and viscometry. The corresponding xerogels are photoconductive. Importantly, the wavelength dependence of the photoconductive films is different to that of the films formed from the perylene bisimide alone. Transient absorption spectroscopy of the xerogels reveals changes in the spectrum of the PBI on the picosecond timescale in the presence of stilbene with a PBI radical anion being formed within 10 ps when the stilbene is present. The ability to form the PBI radical anion under visible light leads to the enhanced spectral response of the multicomponent gels. These systems therefore have potential as useful visible-active optoelectronics.

## Abstract 2

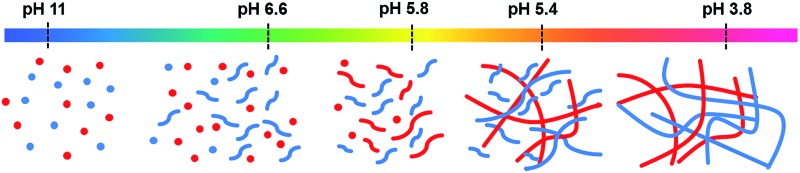
Self-sorting between n-type and p-type gelators results in effective visible-active photoconductive xerogels.

## Introduction

**Fig. 1:**
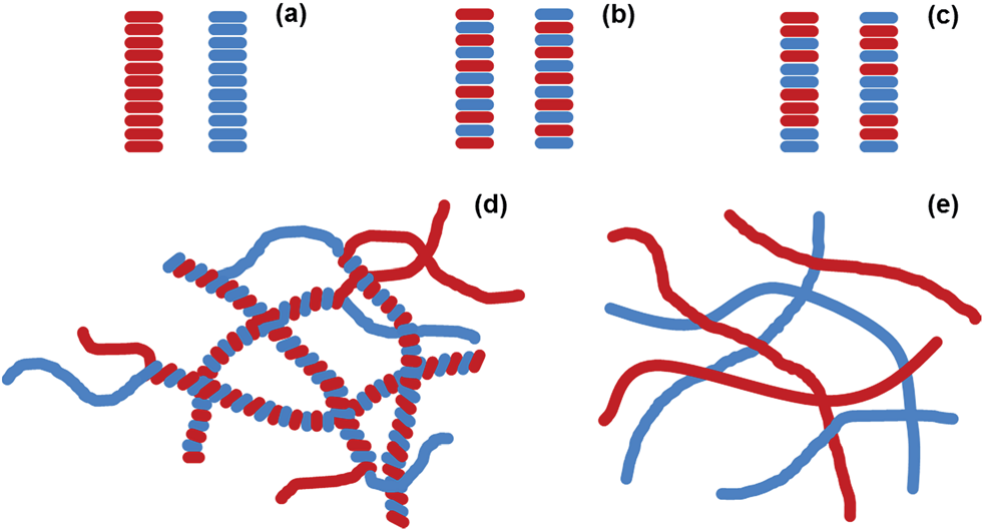
Cartoon showing (a) self-sorting, (b) social co-assembly and (c) random co-assembly of two different gelators. The primary fibres formed in (a) can then further assemble in many ways. Here, we show two hypothetical ways in which self-sorted fibres (red and blue) could interact. (d) Intimate wrapping; (e) interactions only occur at a small number of specific points.

An exciting potential method to generate self-assembled organic photovoltaics (OPV) is to use self-sorted low molecular weight gelators (LMWGs).^1–8^ LMWG form self-assembled fibres in solution.^9,10^ These fibres are typically tens of nanometres wide and up to microns in length. By mixing two different type of LMWG, both of which independently form fibres, it is possible to create interesting new materials.^11–15^ Self-sorted gels consist of systems of fibres, where each fibre contains only one of the two possible gelators (Fig. 1a). These contrast the possibility of co-assembled gels, where each fibre contains each of the two gelators (Fig. 1b and c). Self-sorted fibres have potential in self-assembled OPV. Optimum heterojunction structures must contain nanometre-sized domains of each phase in intimate contact. As such, if self-sorted fibres can be prepared and suitably arranged in space, these provide a real opportunity, again differing from the other potential co-assembly types (Fig. 1b and c). For example, Sugiyasu *et al.* have shown that a self-sorted gel can be used to form a self-assembled p–n heterojunction hydrogel;^16^ the heterojunction is formed where the two fibres meet in the entangled network. Conceptually, for a self-sorted system where each fibre is formed from only one of the LMWG present, the fibres could interact in different ways. As two examples, the two different fibre types could wrap around each other intimately, creating a large surface area heterojunction (Fig. 1d). Alternatively, fibres could have little interaction with each and so would only have a p–n heterojunction where the two fibres meet (Fig. 1e).^11^ Having the donor and acceptor material too close to each other prevents long range order and efficient electron transfer through the material so the second type of interaction would be more preferable.

For most mixtures of LMWG, it is difficult to predict and control whether self-sorting or mixing will occur.^11^ Most examples are based on organogelation (where the gel is mainly an organic solvent) and essentially rely on differences in solubility of each LMWG and differences in the gelation temperature.^12^ A more elegant approach would be to be able to design the assembly process from first principles. We have recently shown that we can utilise a slow, reproducible pH change in water coupled with differences in p*K*_a_ of terminal carboxylic acids to design and control self-sorted hydrogels.^17–19^ We used this approach to prepare self-sorted gels with different rheological properties. We have also found that this approach can be used to prepare gels with independent fibrous networks and that we can selectively remove one network post-gelation using a light-responsive LMWG.^19^

For specific applications such as OPV, we need suitable LMWG that are based on molecules with suitable electronic properties. Ideally, we would want a high interface between p-type LMWG and n-type LMWG. It is also necessary to generate structures with suitable exciton diffusion lengths, as well as suitable spatial arrangement, distribution, and alignment of the self-assembled fibres; all of these parameters need to be controlled over the length-scale of the device. Indeed, for applications in electronics, the packing within the fibres and distribution of fibres in space are expected to have a direct impact on the final conductivity.

There are many reports of perylene bisimides (PBIs, also called perylene diimides, PDIs) as effective low molecular weight gelators.^20–26^ PBIs are effective n-type materials and we have shown that UV-active photoconductive films could be formed from both a dried solution or a dried gel.^27^ PBIs have been used in two component gels previously. As noted above, Sugiyasu *et al.* designed a system using PBI- and thiophene-based gelators.^16^ They used a heat-cool method to prepare a self-sorted organogel using chloroform. Wicklein *et al.* have mixed a PBI with a hole conducting polymer and generated a donor–acceptor heterojunction.^28^ Martín and co-workers co-assembled a PBI and a tetrathiafulvalene into a hydrogel using opposing charges on the molecules.^29^ They reported that the co-assembled structures give long lived separated charge states due to suppressed recombination. Another organogel based system utilised PBI and trithienylenevinylene LMWGs, again using a heat-cool method for self-assembly.^2^ Related work has been carried out with PBIs and oligothiophenes,^30^ PBIs and oligophenylvinylenes,^31^ and with PBIs and carbazoles.^32^ Many of these approaches have been successful to some degree, but there is clearly still a need for improved control over the assembly of the structures.

Here, we focus on designing a two-component LMWG-based system utilising a pH triggered self-assembly to form a self-sorted hydrogel. Our aim here is to exploit our self-sorting approach to prepare a system with an interface between fibres that allows us to modify the properties of a PBI-based gelator. Our system allows the point at which assembly occurs to be pre-determined. We use a photoactive PBI-based gelator as the n-type component to act as an electron acceptor which is mixed with a second p-type gelator which is anticipated to be a suitable electron donor. On drying, a photoconductive xerogel is formed. We show that the presence of the second component significantly affects the properties of the xerogel as compared to the single component system.

## Results and discussion

**Fig. 2:**
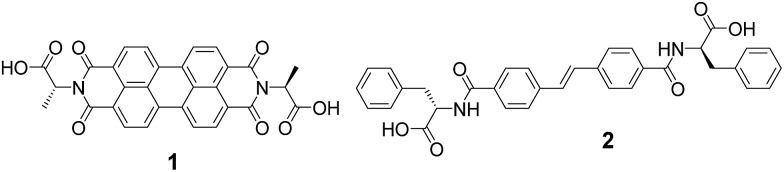
Molecular structures of gelator-**1** and gelator-**2**.

The PBI-based gelator (**1**, Fig. 2) is an n-type material. Electrochemical measurements in solution at pH 9 (Fig. S1†) indicate the PBI/PBI˙^–^ reduction occurs at *ca.* – 0.55 V_Ag/AgCl_ in solution, whilst **1** is oxidised at *ca.* +0.6 V_Ag/AgCl_. We chose a stilbene-based gelator (**2**, Fig. 2) as a potential donor as electron transfer from stilbenes to photoexcited PBIs has been shown to be possible previously^33,34^ and in line with these reports we find a broad redox feature from +0.4 to +0.8 V_Ag/AgCl_ that could potentially act as an electron donor to a photoexcited PBI. In solution at pH 9 significant aggregation of both **1** and **2** occurs which may give rise to the poorly defined electrochemical features. We have previously reported the self-assembly and gelation ability of the single component gels formed from **1** (ref. 27 and 35) and **2**.^19^ Briefly, for **1** and **2** at high pH, the carboxylic acids are deprotonated. This is sufficient to allow self-assembly in water. The apparent p*K*_a_ of the carboxylic acids is higher than might be expected, as we and others have shown for many related gelators.^36–38^ Gelator-**1** forms worm-like micelles at high pH and gels at low pH.^27^ Drying either the solution or the gel phase results in a photoconductive film. Despite the high absorbance at 450–550 nm of the PBI core, the films are only conductive when irradiated at 365 nm; we have recently explained this as being due to the need for more energy than expected to split the photogenerated exciton into a hole and an electron.^27^ Gelator-**2** can also be dispersed effectively at high pH and forms gels at low pH.^19^ In both cases, the gels are the result of the self-assembly of the gelators into fibrous networks.

### Self-sorted donor and acceptor gelator systems

Initially, we focus on the self-assembly of a mixture of **1** and **2**. In two-component systems, there are clearly many permutations for the self-assembly (Fig. 1).^11^ The final material properties will depend on how the two components assemble and so it is critical that we understand the process.^11^ Such systems are hard to characterise and it is difficult to prove self-sorting with only one technique. As such, here we focus on a suite of techniques to first understand the assembly of **1** and **2**. Clearly in such two-component systems, there is a huge parameter space available; for example, it is possible to vary the concentration of each gelator, the rate of assembly, the temperature amongst others. It is well known that the final material properties of even single component gels are very dependent on the process of assembly.^39^ Here we have focused on gaining a comprehensive understanding of one specific set of conditions and hence how the presence of **2** under these conditions affects the photoconductivity of **1**. Whilst this may not represent the optimised conditions, we believe that this will allow us to understand whether or not it is possible to utilise a self-sorted system to affect photoconductivity.

A solution at high pH of gelator-**1** at 10 mg mL^–1^ was added in equal volume to a solution of gelator-**2** at 10 mg mL^–1^ to give a solution at a total concentration of gelator of 10 mg mL^–1^ (5 mg mL^–1^ of each gelator). 10 mg mL^–1^ of glucono-δ-lactone (GdL) was then added to slowly lower the pH to around 3.8 and the sample left to stand overnight to give a self-supporting gel (gel-**1**,**2**). We have previously shown that the slow hydrolysis of GdL to gluconic acid^40^ allows highly reproducible gels to be formed;^37,41^ the slow pH change also allows self-sorted networks to be formed from solutions containing two different LMWG.^15,17–19^ Here, gel-**1**,**2** showed properties typical of LMWGs (Fig. S2, ESI†), breaking at low strain (1.5%) with both the storage modulus (*G*′) and the loss modulus (*G*′′) being independent of frequency (∼9000 Pa and ∼1200 Pa respectively).^42^

Gel-**1**,**2** had properties stronger than gel-**1** but weaker than gel-**2** (Fig. S3, ESI†). We have previously shown that the rheological properties of multicomponent gels are often not simply additive.^18^ To investigate whether the two gelators were self-sorted or co-assembled (Fig. 1),^11^ we utilised a combination of rheological time sweeps and ^1^H NMR spectroscopy, whilst simultaneously measuring the pH. Rheology can be used to measure the formation of a gel and the mechanical properties of the material. When following gelation using ^1^H NMR spectroscopy, the disappearance of peaks corresponding to each gelator can be monitored. When the gelators form fibrous structures, they become NMR-invisible.^43^ Hence, we can correlate the disappearance of a gelator from the NMR spectrum with the formation of fibres. We have previously successfully utilised this combined methodology to test for and demonstrate self-sorting.^17–19^ We exploit the highly reproducible hydrolysis rate of GdL to allow comparison between different samples, although we highlight that *in situ* pH measurements whilst measuring NMR spectra is possible, giving very similar data.^43^ A titration of a solution shows that there are three different plateaus at pH 6.6, 5.8 and 5.4, corresponding to the two p*K*_a_s of gelator **1** and the one p*K*_a_ for gelator **2** (Fig. S4, ESI†). For **1**, we have previously used solutions above the lowest p*K*_a_ to form worm-like micelles and gels.^27^

**Fig. 3:**
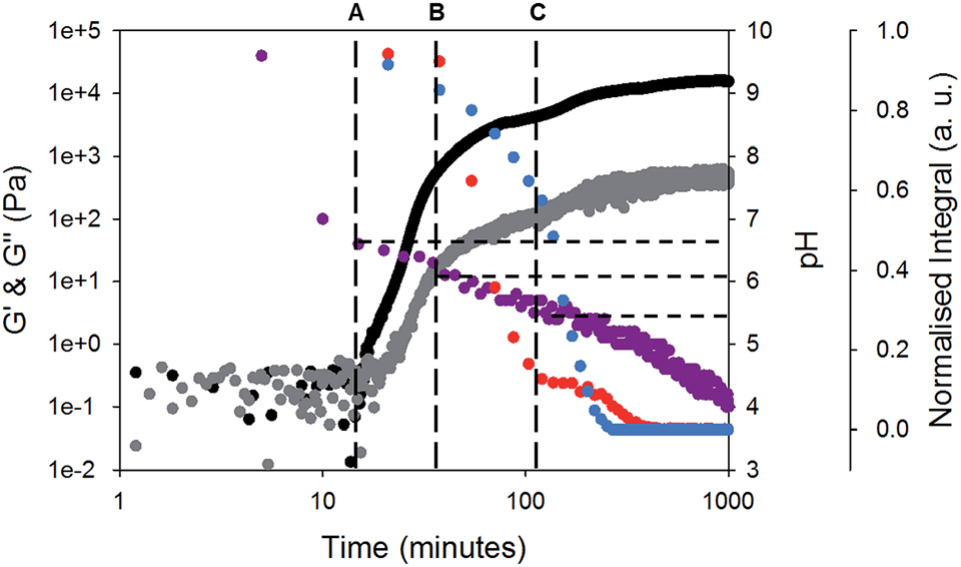
Monitoring gelation of **1** and **2** over time. The change in intensity of peaks from ^1^H NMR spectroscopy during gelation of the CH_3_ peak at 1.70 ppm from gelator-**1** (blue) and the CH_2_ at 3.05 ppm from gelator-**2** (red) are compared to change in pH during gelation of **1** and **2** (purple). The change in *G*′ and *G*′′ over time for gel-**1**,**2** is also shown (black data are *G*′ and grey data are *G*′′), rheological time sweeps were performed at a strain of 0.5%, 10 rad s^–1^ and at 25 °C. For discussion around points A, B, and C, please see main text.

Rheology time sweeps along with the change in pH (Fig. 3) during gelation show that there are multiple stages in the gelation of **1** and **2**. Initially, a free-flowing solution is formed at high pH 9.5. At this point, *G*′ and *G*′′ are both low and similar in magnitude. There is an increase in *G*′ and *G*′′ as the pH reaches 6.6 (point A, Fig. 3). This corresponds to the first p*K*_a_ of **1** (Fig. S3, ESI†). At this point, both gelators are still detectable by NMR spectroscopy. This differs from our previous data, where an increase in *G*′ and *G*′′ correlated with the disappearance of one of the gelators from the NMR spectrum.^17–19^

At point B (Fig. 3), the rate of change of *G*′ and *G*′′ starts to change. Here, the pH is close the p*K*_a_ of **2** (Fig. S4, ESI†). By this point, *G*′ is approximately an order of magnitude larger than *G*′′, showing that a true gel has been formed. At point B, the signals from gelator **2** start to rapidly disappear from the NMR spectra (example NMR spectra are shown in Fig. S5 and S6, ESI†). The integral of **1** does decrease, but at a significantly lower rate as compared to **2**. At point C, where the pH is very close the second p*K*_a_ of **1**, the signal intensity of **1** starts to decrease in the NMR spectra and there is a change in the rate of increase in the rheological data. At point C, there is still 10% of the integral of **2** remaining. As **1** starts to assemble, the integral of **2** does not change for a time, before decreasing again. The gel properties then stabilise as both gelators fully assemble.

When comparing the change in the integrals from each gelator (Fig. 3), it is clear that the integrals of **1** and **2** decrease at different rates, implying that self-sorting is occurring. However, gelator-**1** has a higher p*K*_a_, and so we would expect from our previous work^17,18^ that this would assemble before gelator-**2**. Related work from Tena-Solsona *et al.* has shown a two component system where the order of assembly was also apparently reversed, although this was not explained.^44^ For our system, the pH buffers around the p*K*_a_ of gelator-**1** first. We ascribe this to formation of worm-like micelles by **1** at the first p*K*_a_. We have shown for similar gelators that when worm-like micelles are formed, it is still possible to detect the gelator to some degree using NMR spectroscopy.^45^ This is due to rapid exchange between the gelator in the self-assembled structure and the gelator free in solution (*i.e.* in this case, a structure for which the on–off rate for the gelator is sufficiently quick for gelator-**1** to be NMR-detectable). Hence, we hypothesise that gelator-**1** forms worm-like micelles at its first p*K*_a_ (along with a decrease in NMR signal intensity, but not complete disappearance), followed by the formation of fibres by gelator-**2**, and finally fibres are formed by gelator-**1**.

**Fig. 4:**
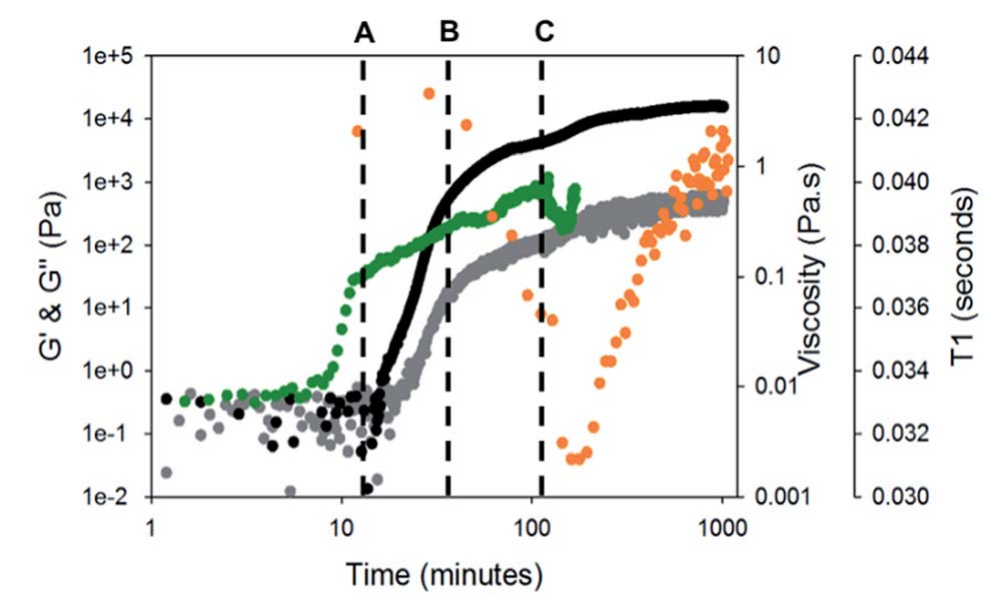
Comparison of change in *G*′ and *G*′′ over time for gel-**1**,**2** (black data are *G*′ and grey data are *G*′′) to change in viscosity (green data) whilst gelling. Orange data are the *T*_1_ relaxation time of ^23^Na^+^ over time. Time sweeps were performed at a strain of 0.5%, 10 rad s^–1^ and at 25 °C. Viscosity measurements were performed at 5 s^–1^. For discussion around points A, B, and C, please see main text.

To test this hypothesis, we measured the viscosity during gelation of a mixture of gelator-**1** and gelator-**2**. If worm-like micelles form, then we would expect that the solution would become more viscous.^46,47^ Indeed, as can be seen from Fig. 4, the solution becomes more viscous before any increase in *G*′ or *G*′′ is recorded (at around 10 minutes, *i.e.* before gelation has begun). This implies that long, entangled structures are formed before gelation has occurred and agrees with the NMR data for gelator-**1**. The viscosity overshoot at around 100 minutes also suggests that these systems could be shear aligned. The formation of structures by the gelators can be further investigated using ^23^Na NMR relaxometry (Fig. 4).^48^ At pH 10, in the absence of GdL, the *T*_1_ relaxation time of ^23^Na^+^ was measured as 42.5 ± 1 ms, well below the *ca.* 57 ms expected for a solution of small molecules.^43^ By ^1^H NMR (Fig. S5, ESI†), the resonances of gelator-**1** are noticeably broadened, while those of gelator-**2** are sharp. These observations point to significant aggregation of gelator-**1** even at pH 10. Upon disappearance of the integrals of gelator-**2** (point B, Fig. 4), the ^23^Na *T*_1_ rapidly falls due to the formation of large, negatively charged structures (Fig. 4) which significantly reduce the mobility of the Na^+^ ions. The *T*_1_ falls again at point C (Fig. 4) before rising again as the pH falls and the negative charge on the assembled fibres is gradually removed.

**Fig. 5:**
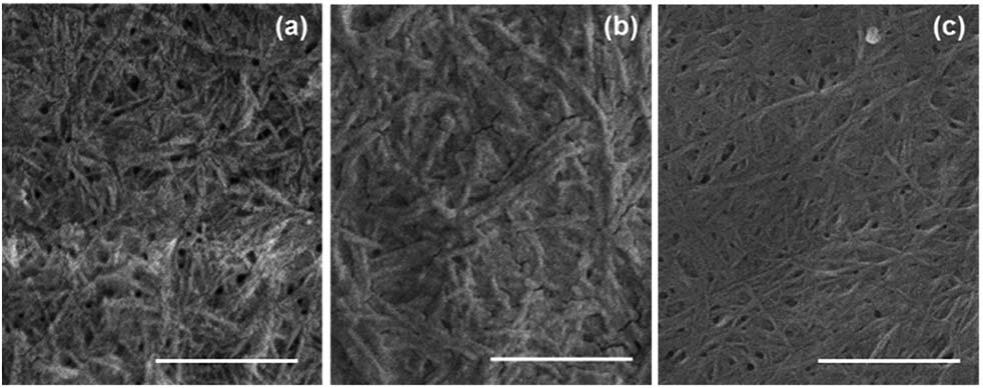
SEM images of (a) xerogel-**1**,**2**, (b) xerogel-**1** and (c) xerogel-**2**. Scale bars represent 500 nm.

Further evidence for self-sorting comes from SEM data. SEM of xerogels (formed by allowing a gel-**1**,**2** to dry in air in a mask) show a uniform domain of fibrous structures. Comparing the xerogels of gel-**1**,**2** with gels formed from either **1** or **2** alone, it is clear that fibrous structures are formed in all cases. However, small differences can be seen in the multicomponent gel (Fig. 5a) compared to the single component gels (Fig. 5b and c). Gel-**1** contains thicker fibres (average 27 nm Fig. S7a ESI†) as compared to gel-**2** (average 17 nm Fig. S7b, ESI†). Fibres with two different thicknesses can be seen in gel-**1**,**2** (Fig. 5a) with a two maxima in the distributions at 14 nm and 27 nm (Fig. S7c, ESI†). These data are indicative of self-sorting as has been shown elsewhere.^49^

**Fig. 6:**
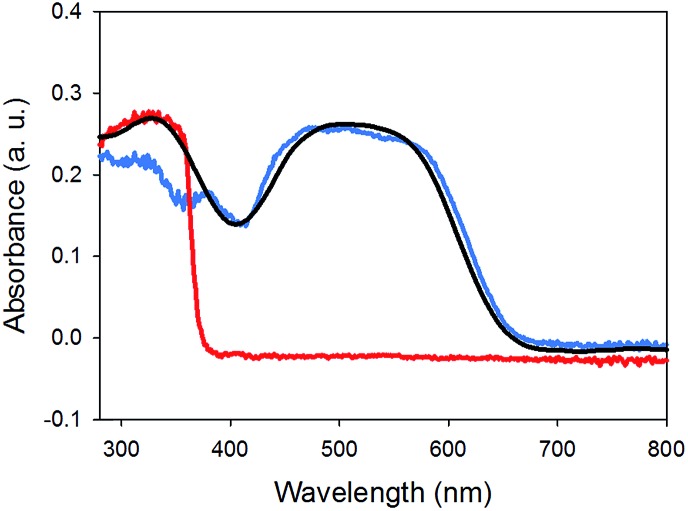
UV-Vis absorption spectra of (a) gel-**1**,**2** (black) compared to gel-**1** (blue) and gel-**2** (red).

Finally, the absorption spectrum of gel-**1**,**2** is essentially the sum of two spectra of a gel of **1** and that of a gel of **2** (Fig. 6). No new peaks appeared, which would be assignable to ground state charge transfer complexes. Hence, similarly to the work of Sugiyasu *et al.*, this is consistent with a self-sorted gel network.^16^

**Fig. 7:**
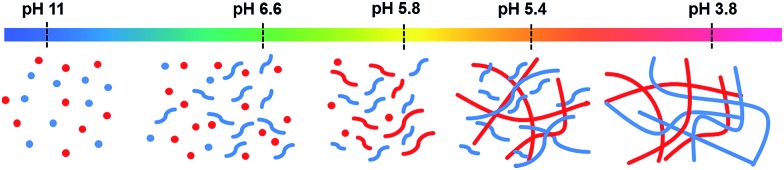
Cartoon of the self-sorting of **1** and **2** at different pH. The flexible short structures represent formation of worm-like micelles, whilst the straighter, longer structures represent fibres.

Looking at these data as a whole, it is clear that the molecules are self-sorting rather than co-assembling. **1** initially assembles into worm-like micelles at its first p*K*_a_. This does not cause an increase in rheological properties, but does increase viscosity and **1** becomes less visible by ^1^H NMR spectroscopy. **2** assembles at its p*K*_a_, the viscosity increases and **2** rapidly disappears from the ^1^H NMR spectra as it gels. **1** then starts to gel at its second p*K*_a_ and so also becomes NMR-invisible; at this point, the rheology increases further. This is shown schematically in Fig. 7.

### Photoresponse of the self-sorted, multi-component system

We have previously shown that xerogels of **1** are photoconductive.^27^ We expect that the photoresponse will be highly dependent on the structure and environment of the materials and on the available charge transfer pathways. Therefore, we explored if inclusion of **2** as a known charge acceptor/donor system^33,34,50^ is able to modify the photoresponse of **1** by comparing xerogel-**1**,**2** with xerogel-**1**. To do this, thin films were prepared using a mask and measured in air. Silver electrodes were placed on both sides of the sample and attached to a potentiostat using copper wires and linear sweeps between –4 V and 4 V were carried out to enable measurement of the change in resistance, and hence the relative conductivity, under illumination (see Fig. S8 and S9, ESI†).

**Fig. 8:**
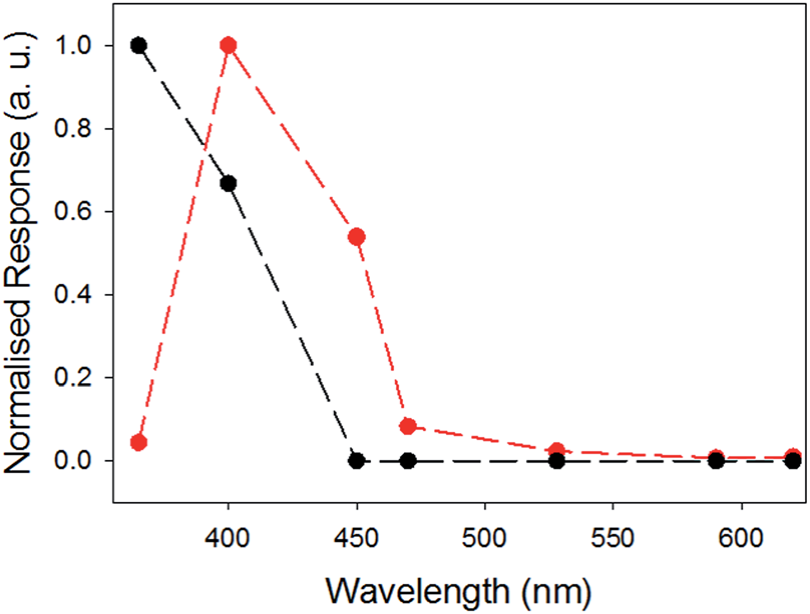
Comparison of normalised photocurrent of xerogel-**1**,**2** (red), and xerogel-**1** (black data) at different wavelengths. The responses were measured at 4 V using a 2 electrode set up, see Fig. S8 and S9.† Data at the different wavelengths were collected for the same samples at different wavelengths after allowing full recovery to baseline before a new measurement was carried out.

The photoconductivity under illumination for xerogel-**1**,**2** was measured and compared to xerogel-**1** (Fig. 8). For xerogels of **1** alone, a large decrease in resistance was observed when the sample was irradiated using a 365 nm LED, as we have shown previously.^27^ Above this wavelength, the photoresponse decreases rapidly. However, for xerogel-**1**,**2**, the greatest response was found when the samples were irradiated at 400 nm. There was little response at 365 nm, the greatest response 400 nm, a significant response at 450 nm and a smaller response at 470 nm. The samples showed no response to wavelengths ≥528 nm. We note that there are differences in the absolute magnitude of the maximum current; this can be ascribed to differences in the degree of alignment and possibly entanglement of the fibres. Whilst important, here we focus on the absolute change in the photoconductivity under light of different wavelengths.

**Fig. 9:**
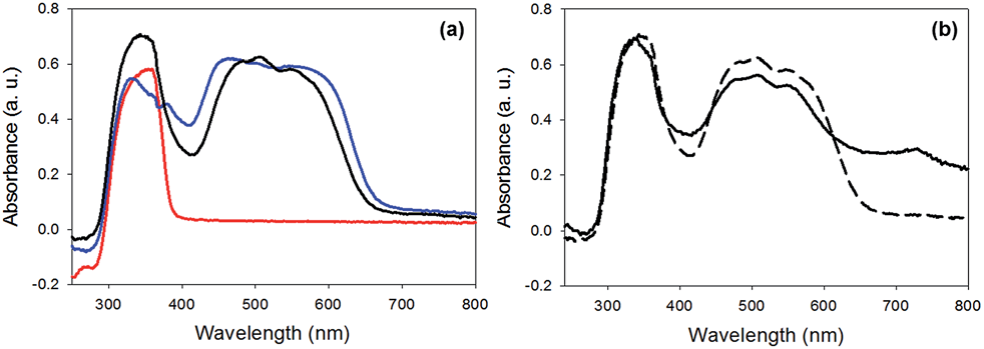
UV-Vis absorption spectra of (a) xerogel-**1**,**2** (black data) compared to xerogel-**1** (blue data) and xerogel-**2** (red data). (b) Xerogel-**1**,**2** before irradiation (dashed data) and after irradiation with a 450 nm LED (solid data).

The photoconductivity can be ascribed to the formation of the radical anion (PBI˙^–^) of **1**, and we have shown in detail elsewhere that the concentration of PBI˙^–^ can be readily measured by UV/Vis spectroscopy and it directly correlates to the level of photoconductivity.^27^ For gels of **1** alone, 365 nm light is required to provide light of an energy greater than the optical gap and the exciton binding energy to form free charge carriers, rather than excitons.^27^ The absorption spectrum of xerogel-**1**,**2** is initially again a sum of the spectra for xerogel-**1** and xerogel-**2**, with peaks for **1** at 450–600 nm and **2** at 300–380 nm (Fig. 9a), in agreement with the data in the gel state (Fig. 6). When xerogel-**1**,**2** was irradiated with 450 nm for 10 minutes, the UV-Vis spectrum showed a new peak at 710 nm (Fig. 9b). This is readily assigned to the formation of the radical anion of **1** through comparison to literature precedent.^27,51,52^ Importantly, irradiating xerogel-**1** with 450 nm light did not result in the formation of the radical anion (Fig. S10, ESI†), explaining the lack of conductivity at this wavelength.

Hence, the self-sorted xerogel-**1**,**2** shows a very different photoresponse to xerogel-**1**. We attribute the lack of response to 365 nm light for xerogel-**1**,**2** to being due to **2** having strong absorption peaks at 365 nm (Fig. 9) leading to competitive absorption by **2**. UV excitation of stilbene is known to lead to *cis*/*trans* isomerisation^53^ including in the gel state.^19^ However, in the xerogel state, we see no evidence for *trans*-to-*cis* isomerisation of **2** (Fig. S11, ESI†). Hence, we ascribe this lower response at 365 nm from the mixed gel to simply competitive absorption of the light by **2** and hence not exciting **1**.

**Fig. 10:**
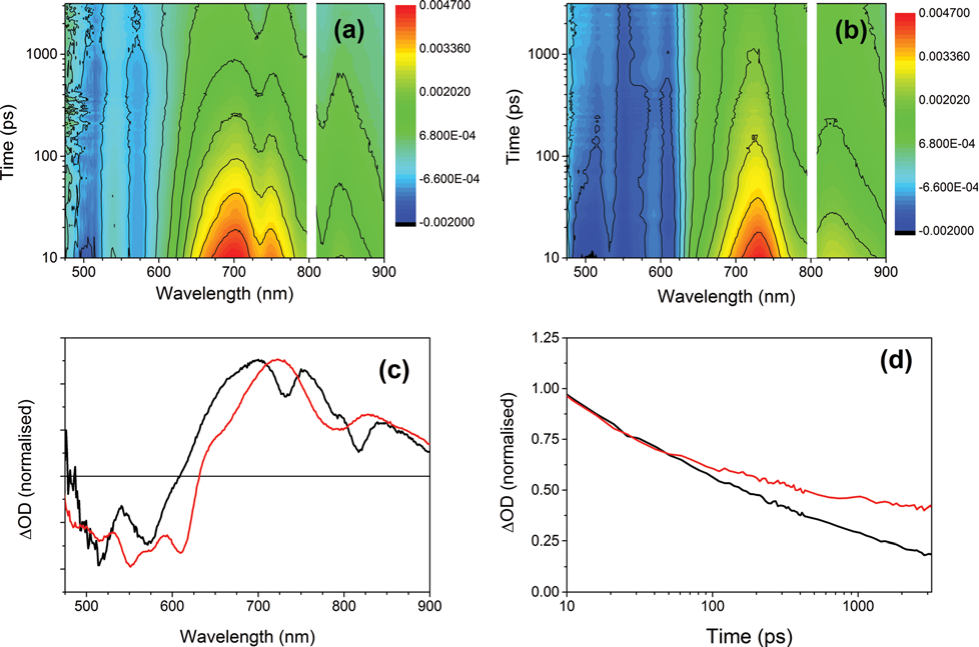
Transient absorption spectra of (a) xerogel-**1** and (b) xerogel-**1**,**2** recorded under 400 nm laser excitation. (c) Normalised absorption spectra of xerogel-**1** (black) and xerogel-**1**,**2** (red) after 1 ns delay time. (d) Normalised decay kinetics of xerogel-**1** (black) and xerogel-**1**,**2** (red) as observed at 750 nm and 725 nm, respectively, attributed to the PBI radical anion.

To further investigate the photoresponse of the multicomponent systems, we employed ultrafast transient absorption spectroscopy, a pump-probe technique that permits time-resolved monitoring of excited state dynamics. Fig. 10 compares the transient absorption spectra of xerogel-**1** and xerogel-**1**,**2** after excitation with 400 nm laser light, a wavelength that selectively excites **1** (Fig. 9). Following the excitation of xerogels of both **1** and **1**,**2** we observed a bleaching of the PBI ground seen as a broad negative signal from 475 to 600 nm, Fig. 10. For xerogel-**1**,**2**, two prominent transient bands are observed (at 725 and 830 nm) showing indistinguishable decay kinetics on the 10 ps to >3 ns timescale. The spectrum of this newly photogenerated species is in excellent agreement with that previously reported for the PBI˙^–^ of **1**,^27,51,52^ indicating that charge separation and formation of the conductive states occurs within 10 ps of excitation of **1** and approximately 40% of the PBI˙^–^ persists beyond 3 ns (compared to the concentration at 10 ps), Fig. 10d. In contrast, excitation of xerogel-**1** leads to a complex transient spectrum with new clear positive features at *ca.* 660, 700, 750, 800, and 840 nm in addition to potential bands at 540 and 610 nm that are overlapped with the ground state bleach, hampering assignment. Kinetic traces recorded at 660, 700, 750, 800 and 840 nm are indistinguishable (Fig. S12†) indicating that the absorption features may be from a single species and this coupled to the lack of agreement with the steady state spectrum of PBI˙^–^ precludes assignment of these features to the reduced form of **1**. Recent transient studies of PBI aggregates assigned a series of broad overlapped spectral features of similar wavelengths to excitonic states formed within 2 ps.^54^

Both the transient and steady state spectroscopic measurements demonstrate that under visible light illumination, a mixed xerogel-**1**,**2** is required for the generation of a long-lived PBI˙^–^ state. The experimental evidence is consistent with **2** acting as an electron donor following excitation of **1**. However, it should be noted that the likely spectral region for a stilbene-based cation (500–600 nm (ref. 50)) is obscured in the transient data by the strong PBI bleaches. The presence of **2** in the mixed xerogel may also aid long-lived charge separation within **1** through changes in the stacking and local structure or by modification of the dielectric environment.^55^

Co-assembly of PBI-based gelators with potential p-type materials has been previously reported.^6,29,30,56–58^ However, to date studies have often used polychromatic light,^32,57^ sacrificial electron donors,^24^ or did not report the individual behaviour of the sub-components,^16^ making it difficult to assess if an active heterojunction was indeed aiding the separation of photogenerated charges.

Our data adds to this growing field of two component self-assembled systems. There are currently only few examples of self-sorted, multicomponent gelled systems where the conductivity is reported. We have shown that we can prepare self-sorted gels from a p-type and an n-type gelator; our pH-triggered approach is predictable from the apparent p*K*_a_ of each component, which is linked to the hydrophobicity,^36^ and so this approach should allow design of other examples. In terms of using these materials in optoelectronic applications, it is critical that visible light can be used, and we have shown here that our approach enables us to successfully shift the photoresponse into the visible.

## Conclusions

Self-sorted gels can be formed by the sequential assembly of two low molecular weight gelators. Unusually, the assembly shown here for a PBI-based gelator and a stilbene-based gelator follows a sequence whereby one gelator initially forms worm-like micelles, followed by the second gelator forming fibres, and then the first gelator forming fibres. Investigation of the photoresponse of the corresponding xerogels indicate that the stilbene-based gelator acts as an electron donor, resulting in a change to the wavelengths at which the system is photoconductive as compared to the perylene-based system alone. The self-sorted network results in a photoresponse at higher wavelengths. We have shown that the presence of the stilbene results in a significant decrease in the rate of the decay of the PBI radical anion. Hence, the self-sorted system has improved properties over the single component PBI. We envisage that our method of producing self-sorted gels will be amenable to a range of n- and p-type gelators and we are actively pursuing this concept.
