# Supplementary material for: Self-sorted photoconductive xerogels
Source: Chem Sci. 2016 Jul 1;7(10):6499–505. doi: 10.1039/c6sc02644c (PMC5355952; doi:10.1039/c6sc02644c)
Supplement: Supplementary file 1 [file SC-007-C6SC02644C-s001.pdf]

Electronic Supplementary Material (ESI) for Chemical Science.
This journal is © The Royal Society of Chemistry 2016

Self-Sorted Photoconductive Xerogels

Emily R. Draper,[a] Jonathan R. Lee,[b,c] Matthew Wallace,[a] Frank Jäckel,[b,c] Alexander J. Cowan[a,c] and Dave J. Adams[a,*]

[a] *Department of Chemistry, University of Liverpool, Crown Street, Liverpool, L69 7ZD, U.K.*

[b] *Department of Physics, University of Liverpool, Oxford Street, Liverpool, L69 7ZE, U.K.*

[c] *Stephenson Institute for Renewable Energy, University of Liverpool, Peach Street, Liverpool, L69 7ZF, UK.*

SUPPORTING INFORMATION

Experimental

Materials Gelator-**1** and gelator-**2** were prepared as described elsewhere.[1, 2] All chemicals were purchased from Sigma-Aldrich and used as described. Deionised water was used throughout.

Preparation of Low Molecular Weight Gelator (LMWG) Solutions Gelator-**1** was added to 2 mL of water with an equimolar amount of sodium hydroxide (0.1 M, aqueous) to a concentration of 5 mg/mL. The solution was stirred until all the gelator was dissolved. For gelator-**2**, two molar equivalents of sodium hydroxide were used. For solution- **1,2** each gelator was prepared at 10 mg/mL and then an equal amount of each added together resulting in 1:1 5mg/mL of each gelator solution.

Hydrogel Formation A pH switch method was used to form the hydrogels. Solutions were prepared as above. The solution was then transferred to a vial containing a pre-weighed amount of glucono-δ-lactone (GdL) and shaken gently. The sample was then left to stand overnight to allow gelation to occur. For gelator-**1** and gelator-**2**, 5 mg/mL of GdL was used. For a ratio of 1:1, 1 mL of a 10 mg/mL solution of **1** was added to 1 mL of a 10 mg/mL solution of **2**. This resulted in a 5 mg/mL of each gelator being present and an overall concentration of 10 mg/mL. 10 mg/mL of GdL was then added for gelation.

Preparation of samples on glass slides for photoconductivity measurements and UV-Vis measurements Samples were prepared by dropping 10 μL of the LMWG solution onto a glass microscope slide inside a 3 mm x 3 mm mask and then left overnight to dry in air. When the sample had dried the mask could be removed leaving a 3 mm x 3 mm square. Xerogel samples were prepared by forming gels as described above using GdL inside a 1 mL mould. Once gelation had occurred, the gel was then removed from the mould and approximately 0.05 mL of the gel was removed using a scalpel, placed onto a glass microscope slide, again with a 3 mm x 3 mm mask and

allowed to dry in air overnight. When the sample was dried the mask could be removed and two silver electrodes spaced 3 mm apart were then added. The silver electrodes were made using silver paste which attached copper wires to the glass slide. Epoxy resin glue was placed over the silver electrodes. Again, this was left to dry overnight before measurements.

Preparation of xerogel thin films for TAS measurements Solutions of each sample were prepared described above. As samples needed to be thin and so a different approach was needed. After GdL was added to the solutions and dissolved, 20 µL was removed and spread over a glass microscope slide so that it covered the majority of the slide. This was then sealed in a hydrated chamber to allow gelation to occur and preventing drying out of the film. Gelation could be monitored by the sample in which the solution was taken from. These were left overnight. The sample was then removed from the hydrated chamber and allowed to dry, giving a thin xerogel film.

Photoresponse Measurements were performed using an Autolab Potentiostat operating in a two electrode configuration in the absence of a supporting electrolyte. A 365 nm, 400nm, 450 nm, 470 nm, 528 nm, 590 nm and 628 nm LEDs (LedEngin Inc, LZ1-10U600) with a light source powered by a TTi QL564P power supply operating at 3.9 V were used as a light supply. Dark experiments were performed in an enclosure in air. Linear sweep measurements were recorded from -4 V to 4 V at a scan rate of 0.05 V/s and a preconditioning step at 0.002 V for 2 seconds. The current recorded a 4 V was then used as a value for photoresponse value at each wavelength.

UV-Vis absorption spectroscopy UV-Vis absorption spectra of thin film xerogels were obtained using a Shimadzu UV-2550 UV−Vis spectrophotometer running the UV Probe software, version 2.34 fitted with an integrated sphere attachment. Spectra were measured either up to 800 nm with a scan speed set to medium and using a slit width of 5.0 nm in absorption mode. Samples were prepared as previously mentioned with GdL. This gel was then transferred onto a glass slide and allowed to dry in air overnight in air to form a thin film xerogel.

UV-vis absorption spectra of wet samples at 5 mg/mL were carried out using the same instrument but using a 0.1 mm quartz cuvette. Samples were allowed gel overnight inside the cuvettes before they were measured using the same conditions as before.

Fluorescence spectroscopy Solid-state fluorescence measurements were performed on a Shimadzu spectrofluorophotometer fitted with a solid sample accessory. Spectra were recorded with a 1 nm sampling interval at one 1 nm s^{-1}. Emission spectra were recorded between 490 nm and 690 nm at an excitation of 470 nm. Samples were prepared on glass slides as previously described.

Scanning Electron Microscopy SEM images were obtained using a Hitachi S-4800 FE-SEM. Gels and solutions at high pH were deposited onto glass cover slips which were stuck onto aluminium SEM stubs using sticky carbon tabs and left to dry for 24 hours.

pH Measurements A FC200 pH probe (HANNA instruments) with a 6 mm x 10 mm conical tip was used for pH measurements. The stated accuracy of the pH measurements is ±0.1. For pH measurement during gelation pH was recorded every minute until a gel was formed. The temperature was maintained at 25 °C during the titration by using a circulating water bath. For "apparent" pK_a measurements pH were recorded after each addition of HCl and a stable value was reached. To prevent a gel forming, the solutions were gently stirred using a stirrer bar, so keeping the sample a liquid during the "titration" process. The plateaus of the pH indicates the two pK_a of this gelator. Temperature was kept at 25 °C during the titration by using a water bath.

Rheological Measurements Dynamic rheological and viscosity measurements were performed using an Anton Paar Physica MCR101 and MCR301 rheometer. A cup and vane measuring system was used to perform frequency and strain sweeps. A cone and plate measuring system was used to perform viscosity measurements and gelling under shear. A parallel plate

measuring system was used for time sweeps. For frequency and strain tests, 2 mL of the gels were prepared in 7 mL Sterilin vials and left for 24 hours at room temperature before measurements were performed. For viscosity measurements, samples were prepared at high pH as previously mentioned. For time sweeps and gelling under constant shear, the gels were prepared in a vial and transferred onto the bottom plate. All experiments were performed at 25 °C.

Frequency sweep: Frequency scans were performed from 1 rad/s to 100 rad/s under a strain of 0.5 %. The shear modulus (storage modulus (G') and loss modulus (G")) were read at 10 rad/s. These measurements were done within the viscoelastic region were G' and G" were independent of strain amplitude.

Strain sweep: Strain scans were performed from 0.1 % to 100 % with a frequency of 10 rad/s. The critical strain was quoted as the point that G' starts to deviate for linearity and ultimately crosses over the G", resulting in gel breakdown. From this data, a strain of 0.5 % used for measuring the frequency sweep was in the viscoelastic region.

Viscosity measurements: Viscosity measurements were performed using a 25 mm cone and plate. Around 0.2 mL solutions were transferred onto the plate for measurement after GdL was added. The viscosity of each solution was recorded under the rotation shear rate of 5 s^{-1} during gelation.

Time sweeps: Time sweeps were performed with a 50 mm plate with a plate gap of 0.8 mm. Tests were performed at an angular frequency of 10 rad s^{-1} and with a strain of 0.5 %. The top plate was flooded with mineral oil to prevent the sample from drying.

Fibre Distribution Measurements Measurement of fibre widths were performed using ImageJ image analysis software. Images used for measurements were collected by SEM. A total of 90 measurements on each image were used to create the distribution curves.

Nuclear Magnetic Resonance Spectroscopy The NMR time series of Figures 2 and 4 was collected on a Bruker AvanceII 400 MHz wide bore spectrometer. All measurements were performed off lock in H_2O at 298 K. ^1H spectra were acquired with the double-echo W5 WATERGATE sequence of

Liu et al. (Bruker pulse program library ZGGPW5).[3] The delay between successive pulses in the selective pulse train was set at 125 μs corresponding to a 4000 Hz separation between the null points. 8 scans were acquired with a signal acquisition time of 2 s and a relaxation delay of 8.1 s, giving a total acquisition time of 80 seconds. Integrals of gelators **1** and **2** were obtained relative to sodium methanesulfonate (1 mM) added as an internal chemical shift reference. The integrals on Figure 2 have been normalised to their values measured at pH 10. ^{23}Na T_1 relaxation times were measured using the inversion-recovery pulse sequence. The inversion recovery time, t, was varied between 1 and 300 ms in 8 steps. 32 scans were collected in 6144 points with a 100 ppm sweep width and a relaxation delay of 0.1 s, giving an acquisition time of 2 minutes. The ^{23}Na π/2 pulse was calibrated at high pH prior to the addition of GdL using the Bruker optimisation procedure POPT and was found to be 32 μs in duration. ^{23}Na T_1 relaxation times were obtained by fitting the experimental curves to equation S1:

$$I = I_0\left[1 - P.exp\left(-{}^{t}/_{T_1}\right)\right] \qquad \text{(S1)}$$

where I is the measured signal intensity, t is the time since initial excitation of the magnetization, P ≈ 1.9 and I_0 is a fitting parameter representing the signal intensity at t = 0. Excellent fits ($R^2 > 0.997$) were obtained to Equation S1 in all cases.

Electrochemical Measurements Electrochemical studies were carried out on aqueous solutions (5 mg/ml, pH 9, 0.1 M NaCl) of **1** and **2** using a glass carbon working electrode, platinum counter electrode and a silver/silver chloride reference (BASI). All solutions were thoroughly purged with either Argon or Nitrogen for 20 minutes and then kept under a blanket of the same gas. Square-wave voltammograms were recorded using an Emstat potentiostat (PalmSens).

Transient absorption spectroscopy was performed with a laser system based on an ultrafast regenerative amplifier, optical parametric amplifier and second harmonic generator (Light Conversion: Pharos, Orpheus and Lyra)

which generates excitation pulses of ~200 fs at the pump wavelength. Transient absorption spectra were then recorded using a white light continuum and a Helios spectrometer (Ultrafast Systems).

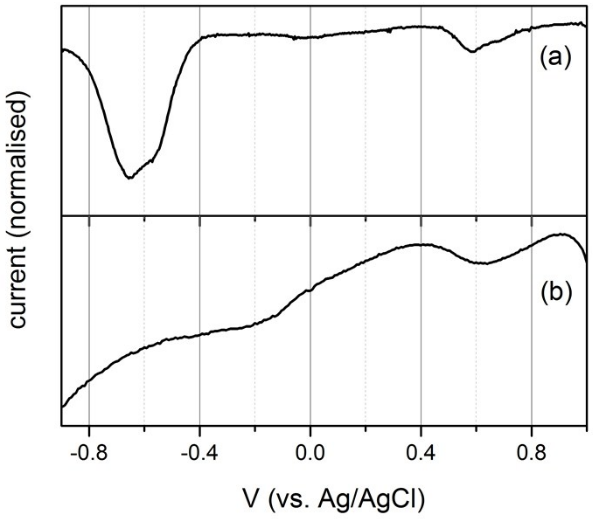

Figure S1. Square-wave voltammetry of (a) **1**, (b) **2** in aqueous solution (0.1 M NaCl, pH 9) recorded under an argon atmosphere using a glassy carbon working electrode at 40 mV s^{-1} (10 H_z, 4 mV step, 10 mV amplitude).

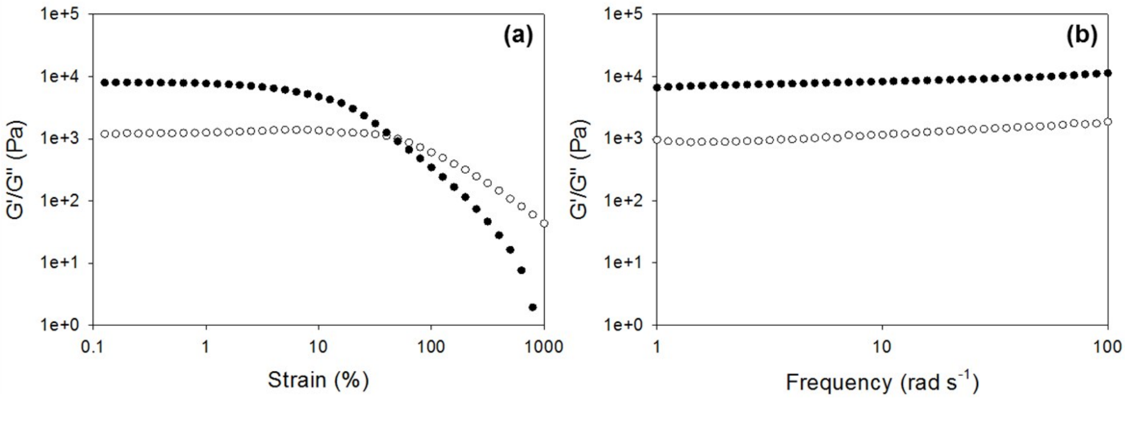

Figure S2. Rheology of gel-**1**,**2** (a) strain sweep performed at 10 rad/s and (b) frequency sweep performed at 0.5 % strain. Both tests were performed at 25 °C. Full circles represent G' and open circles represent G".

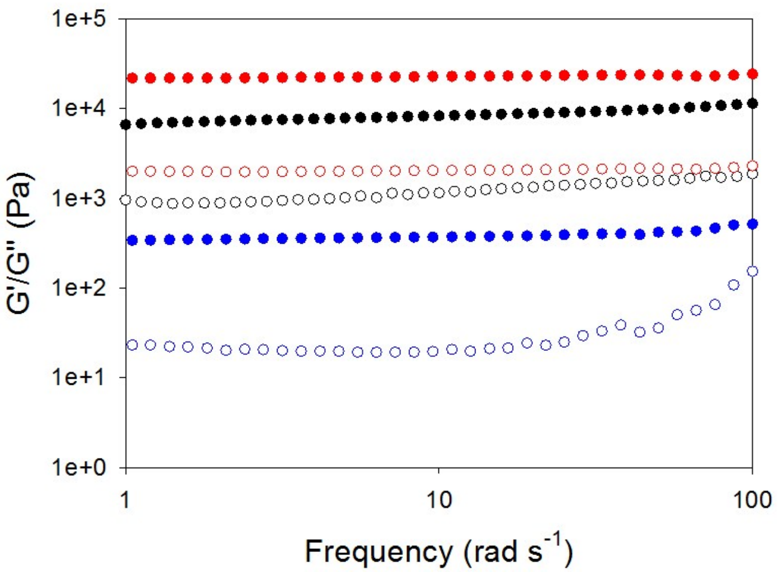

Figure S3. Frequency sweeps of gel-**1** (blue data), gel-**2** (red data) and gel-**1**,**2** (black data). Tests were performed at a strain of 0.5 % strain and at 25 °C. Full circles represent G' and open circles represent G".

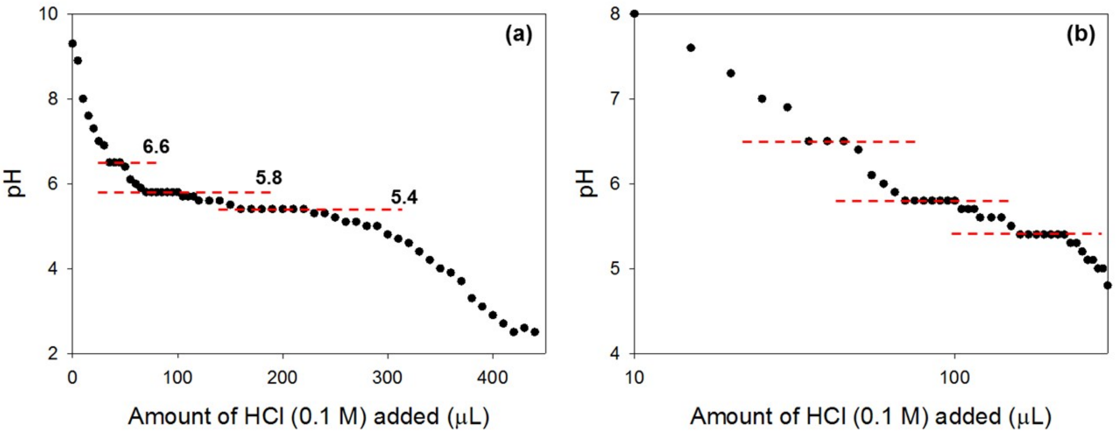

Figure S4. Change in pH upon the addition of 0.1 M HCl in a solution of **1**,**2** (a) on a linear scale and (b) on a log time scale. Dashed lines have been added to mark the plateaus.

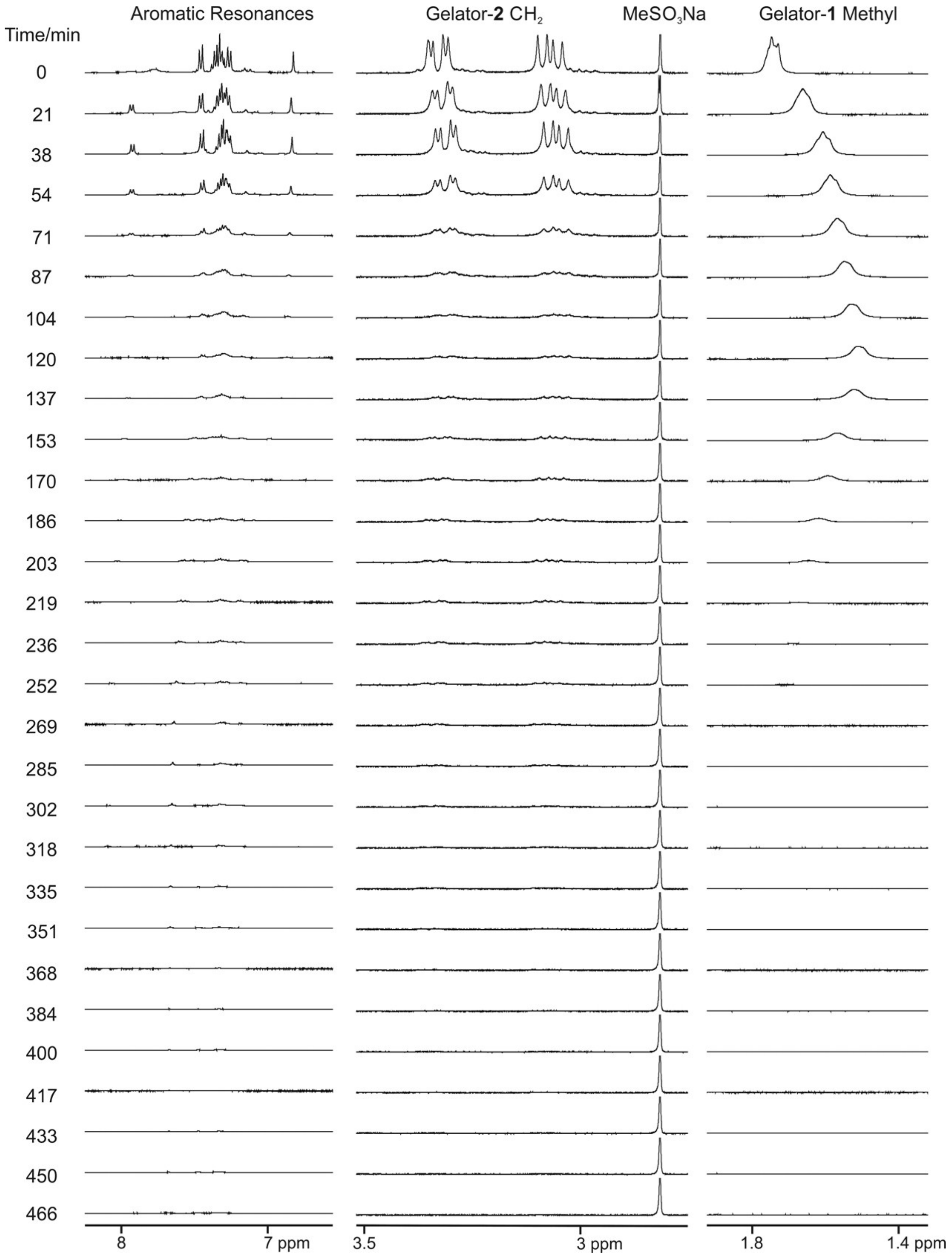

Figure S5. ^{1}H NMR spectra of sample used to create Figure 3 at times indicated since the addition of GdL to a solution of gelators **1** and **2** at pH 10. The spectrum at t = 0 was recorded prior to the addition of GdL.

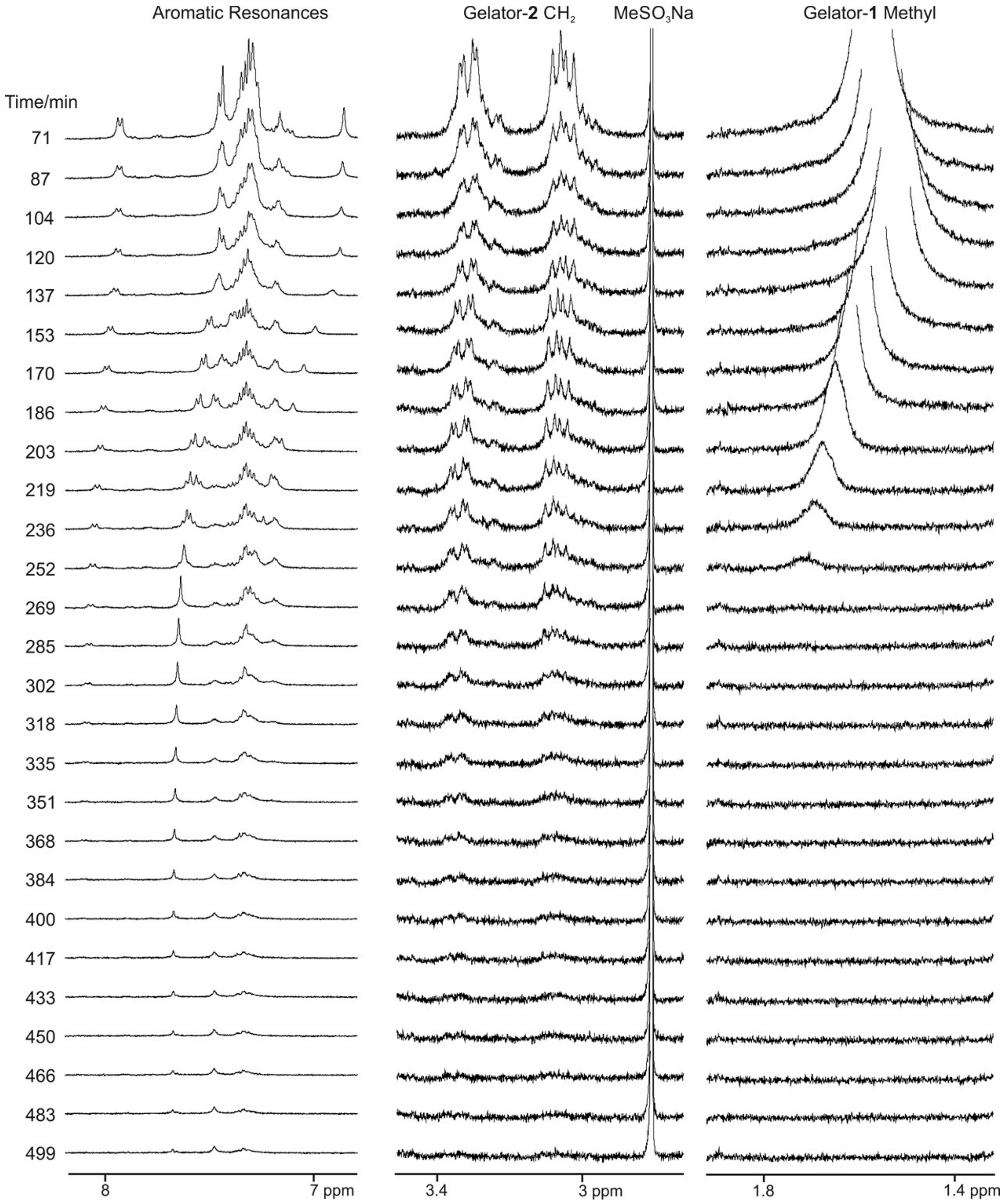

Figure S6. NMR spectra of Figure S5 between 71 and 499 minutes after the addition of GdL to a solution of gelators **1** and **2** at pH 10. The intensity of the spectra have been scaled so that the weak resonances present at later time points are more clearly observable.

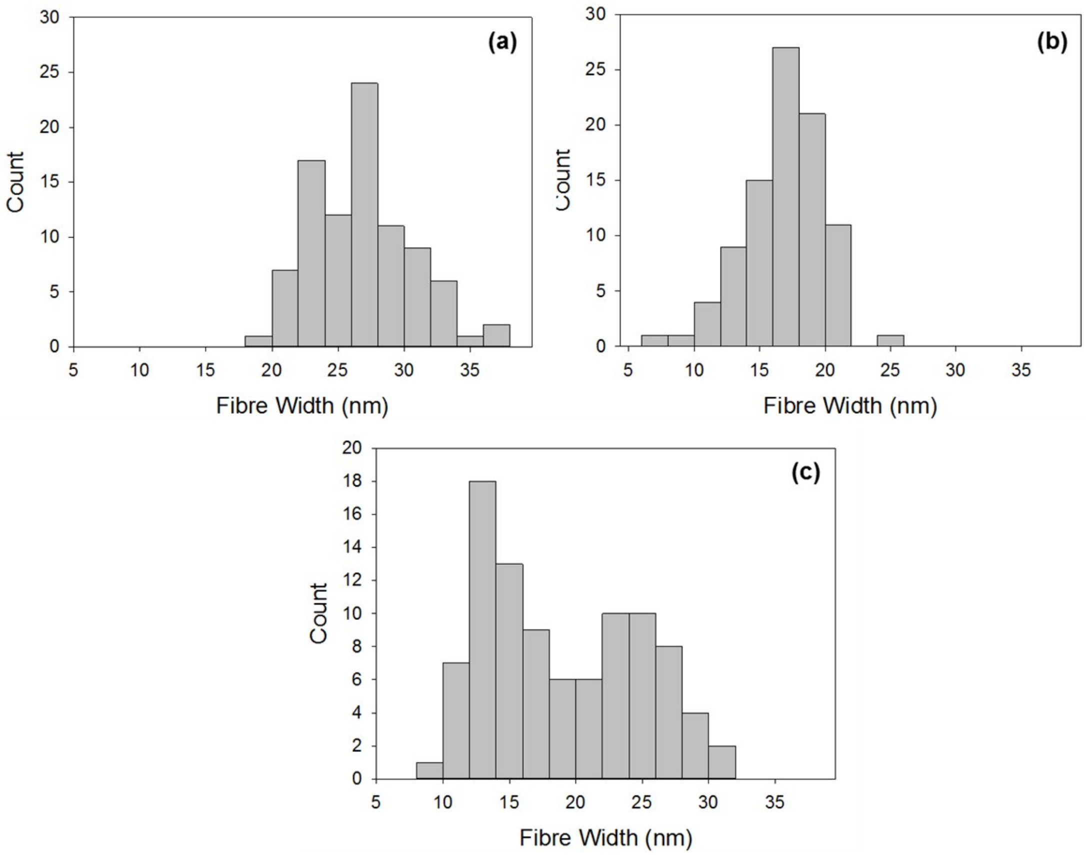

Figure S7. Fibre distributions from 90 measurements using ImageJ image analysis software. SEM images from Fig. 5 were used for analysis. (a) Xerogel-**1**, (b) xerogel-**2** and (c) xerogel-**1**,**2**.

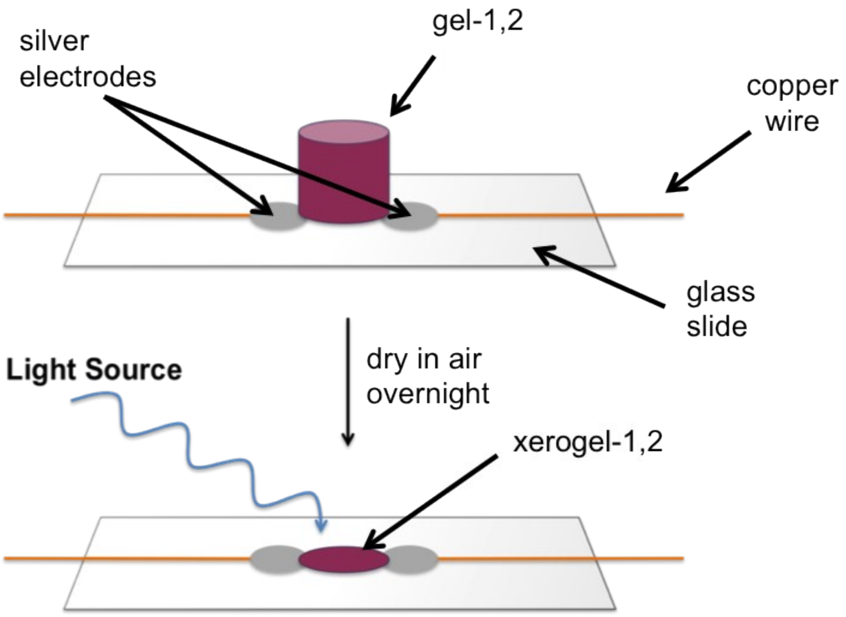

Figure S8. Cartoon showing the set up for conductivity measurement of xerogels.

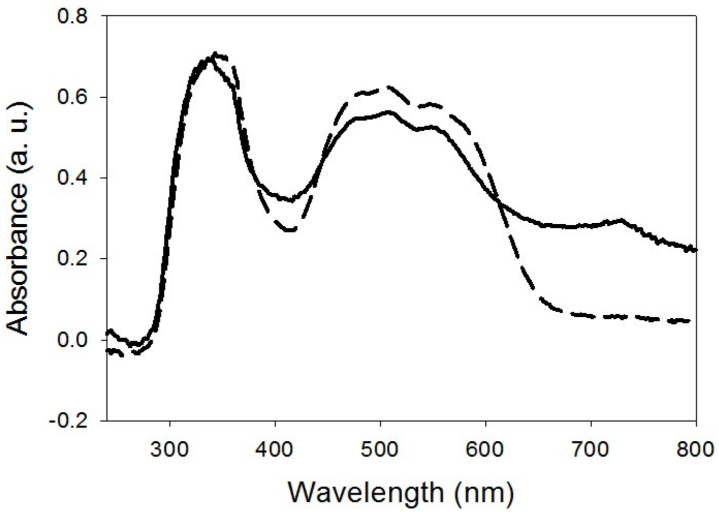

Figure S9. Example data for xerogel-**1**,**2** illuminated at 450 nm (black line) as compared to data collected in the dark (dashed line).

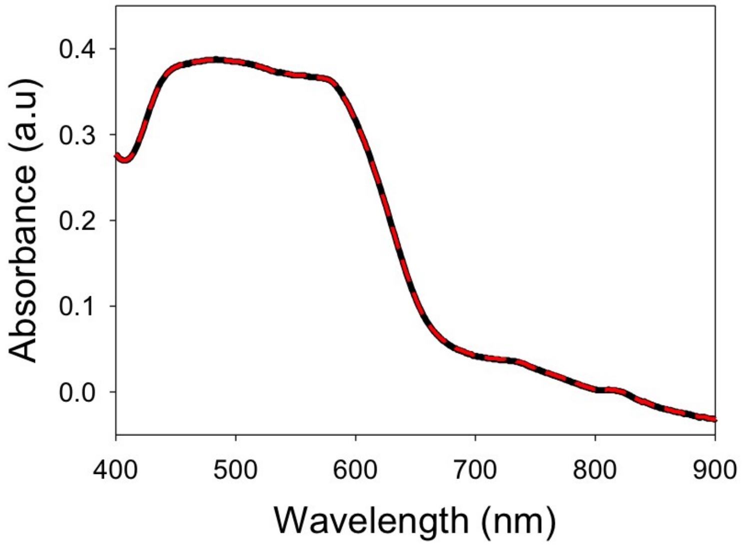

Figure S10. UV-vis spectra of xerogel-**1** (black data) and after irradiation with a 450 nm LED (red dashed data).

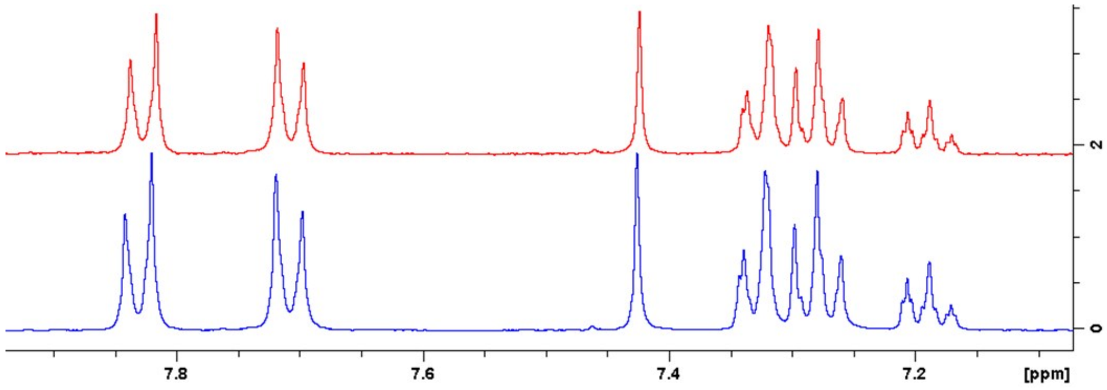

Figure S11. ^1H NMR spectra of xerogel-**2** in DMSO-d_6 between 7.9 ppm and 7.1 ppm before irradiation (blue spectra) and after irradiation (red spectra) showing no trans-to-cis isomerisation has occurred upon irradiation of the dried film.

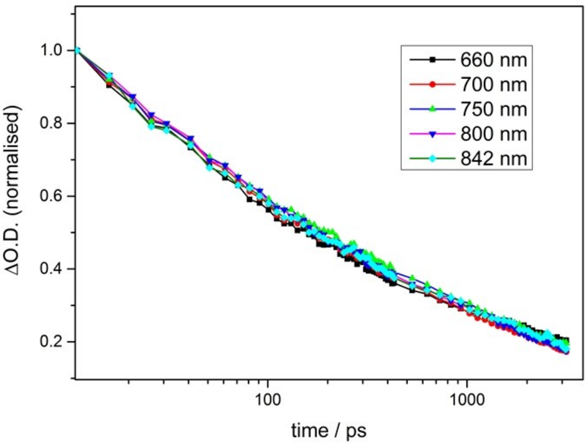

Figure S12. Transient absorption kinetics recorded at the wavelength indicated following 400 nm excitation of xerogel-**1**.

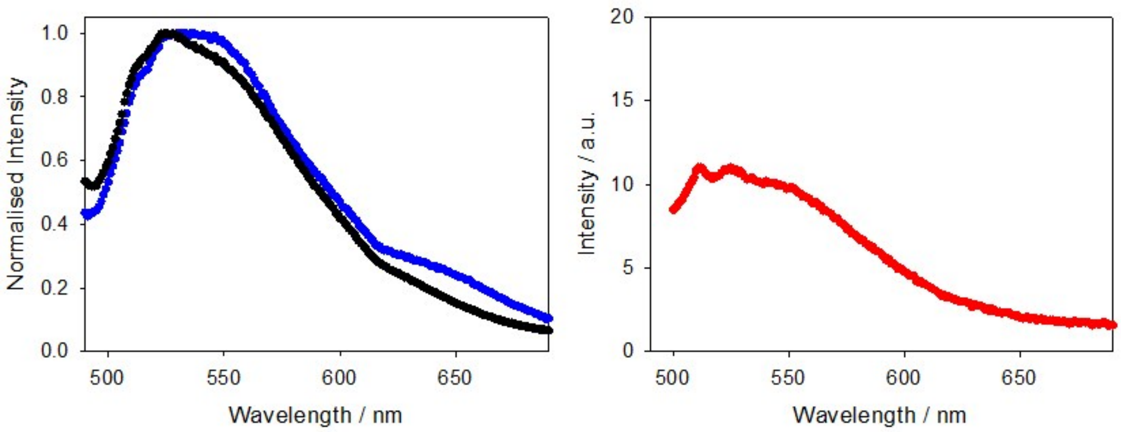

Figure S13. (Left) Normalised emission spectra of xerogel-**1** (blue data), and xerogel-**1**,**2** (black data) at an excitation of 470 nm. (Right) Emission spectra of xerogel-**2** at an excitation of 470 nm. Quenching of emission has been used previously to indicate electron transfer in many systems. In our system emission from the singlet state (λ_{max} *ca.* 545 nm) of the PBI core of (**1**) is already significantly quenched due to aggregation within the gel following visible light (470 nm) excitation. A weaker red-shifted second feature seen at *ca.* 650 nm is tentatively assigned to emssion from an excimer state which can occur upon aggregation.[4] It is difficult to control absolute film thickness for the different xerogels making quantitative comparison between xerogel **1** and the mixed xerogel of **1** and **2** difficult. Therefore, we have provided data on both systems as normalised spectra. The mixed xerogel of **1** and **2** shows a strong emission feature at *ca.*545 nm that is very similar to that of **1** alone. It is however notable that the emission at *ca.* 650 nm is largely quenched in the mixed xerogel. This may indicate that the excimer state of PBI **1** is being quenched by electron transfer from **2** in the mixed gel. This would be in line with our transient spectroscopy study which provides more direct evidence that the heterojunction enables the formation of the $PBI^{\cdot -}$ which is proposed to be due to electron transfer from **2**.

References

1. E. R. Draper, J. J. Walsh, T. O. McDonald, M. A. Zwijnenburg, P. J. Cameron, A. J. Cowan and D. J. Adams, *Journal of Materials Chemistry C*, 2014, **2**, 5570-5575.
2. E. R. Draper, E. G. B. Eden, T. O. McDonald and D. J. Adams, *Nat Chem*, 2015, **7**, 848-852.

3. M. Liu, X.-a. Mao, C. Ye, H. Huang, J. K. Nicholson and J. C. Lindon, *Journal of Magnetic Resonance*, 1998, **132**, 125-129.
4. B. Rybtchinski, L. E. Sinks and M. R. Wasielewski, *The Journal of Physical Chemistry A*, 2004, **108**, 7497-7505.
